# Identification of Phytochrome-Interacting Factor Family Members and Functional Analysis of *MdPIF4* in *Malus domestica*

**DOI:** 10.3390/ijms21197350

**Published:** 2020-10-05

**Authors:** Peng-Fei Zheng, Xun Wang, Yu-Ying Yang, Chun-Xiang You, Zhen-Lu Zhang, Yu-Jin Hao

**Affiliations:** State Key Laboratory of Crop Biology, College of Horticulture Science and Engineering, Shandong Agricultural University, Tai’an 271000, China; zhengpengfei96@163.com (P.-F.Z.); wx20145015@126.com (X.W.); 17863807915@163.com (Y.-Y.Y.); youchunxiang@sdau.edu.cn (C.-X.Y.)

**Keywords:** phytochrome-interacting factor, gene expression analysis, protein localization, ABA sensitivity, transcription activation activity

## Abstract

Phytochrome-interacting factors (PIFs), members of the basic helix-loop-helix transcription factor family that have been extensively investigated in *Arabidopsis thaliana*, play essential roles in plant growth and development. However, *PIF* members have not been systematically investigated in apples, a worldwide perennial woody crop of economic importance. Here, seven *PIF* genes were identified from the *Malus × domestica* reference genome. Chromosomal locations, gene structures, and phylogenetic relationships of these members were analyzed. Analysis of cis-acting elements in promoter regions of *MdPIF* genes indicated that various elements were related to light, abiotic stress, and plant hormone responsiveness. Subsequently, subcellular localization and transcriptional activity analysis revealed that MdPIFs were typical nuclear transcription factors with transcriptional activation ability. Expression analysis demonstrated that *MdPIF* genes had different gene expression patterns for various abiotic factors. Moreover, overexpressed *MdPIF4* reduced the sensitivity of apple calluses to abscisic acid (ABA). Our work lays foundations for further investigation of PIF functions in plant growth and development in apples.

## 1. Introduction

Light is one of the most important environmental factors affecting plant growth and development [1]. Due to differences in light spectrum, intensity, direction, and photoperiod, a series of photoreceptors, including phytochrome, cryptochrome, and phototropin, have evolved to adapt plants to different light conditions [1,2,3]. Phytochrome is an important class of photoreceptors that perceives and responds to red and far red light, and it participates in the entire growth and development process, from seed germination to vegetative growth and maturity of plants [4,5]. To date, five phytochromes (phyA–phyE) have been identified in *Arabidopsis*, and they have two photo-interconvertible forms [6,7]. When exposed to red light, the conformation of phytochromes changes allosterically from the inactive red light-absorbing (Pr) form to the active far red light-absorbing (Pfr) form. The inactive Pr form is located in the cytosol, whereas the active Pfr form resides in the nucleus [6,7]. Upon translocation into the nucleus, phytochromes in active form interact with multiple factors to regulate the transcription of downstream target genes and mediate subsequent orchestrated photoreactions [8,9,10,11].

Phytochrome interacting factors (PIFs) are one of the most important phytochrome interacting partners, and they primarily act as negative regulators of photomorphogenesis in response to light, and maintain skotomorphogenesis in darkness [8,12]. Currently, eight PIFs (AtPIF1–AtPIF8) have been identified in *Arabidopsis* [9], and AtPIF3 was originally identified as a phytochrome-interacting protein using AtphyB as bait [13]. As basic helix-loop-helix (bHLH) transcription factor family members, all AtPIFs contain the bHLH domain, which plays a key role in the formation of homo- and heterodimers of AtPIFs [9]. In addition, the active phyB-binding (APB) motif is present in all AtPIFs, whereas the active phyA-binding (APA) motif only exists in AtPIF1 and AtPIF3 [8,14,15].

In addition to being a negative regulator of photomorphogenesis in response to light [8,16,17], PIFs have also been demonstrated to regulate many other pathways, including anthocyanin biosynthesis, thermomorphogenesis, hormone signaling, and responding to biotic and abiotic stresses by interacting with multiple cellular molecules [18,19,20,21]. For example, maize (*Zea mays*) *ZmPIF1* and *ZmPIF3* could improve the drought tolerance of rice by inducing stomatal closure [22,23]. AtPIF1 interacted with AtABI5 (abscisic acid insensitive 5), a basic leucine zipper (bZIP) transcription factor that is involved in the abscisic acid (ABA) signaling pathway, to regulate endogenous ABA and ABA signaling pathways [24,25]. Furthermore, hypocotyl growth of the *pif4* mutant is insensitive to high temperature (29 °C), suggesting the key role of *AtPIF4* in plant thermoresponsive growth [26]. Moreover, *AtPIF4* was also reported to accelerate flowering by directly activating *AtFT* (*flowing locus T*) expression in a temperature-dependent manner [27]. Therefore, PIFs are considered to be a hub in the integration of environmental and hormonal signaling pathways [28].

Apple (*Malus domestica*) is an important commercial fruit crop, and its production is highly affected by environmental factors, especially light [29,30]. PIFs play a central role in adapting plants to different light conditions; however, identification and functional analysis of *PIFs* in apples have not been systematically investigated, except *MdPIF1* [31]. Here, we identified seven *MdPIF* genes using bioinformatics methods and analyzed their gene structures, chromosomal locations, conserved motifs and the cis-acting elements of their promoter regions. The expression patterns under different stresses were analyzed to better understand the function of apple *PIF* genes. Moreover, overexpressed *MdPIF4* reduced the sensitivity of apple calluses to ABA. Our findings establish a foundation for the further functional investigation of plant PIFs.

## 2. Results

### 2.1. Identification, Chromosome Localization, and Gene Structural Analysis of the MdPIF Genes

PIFs are members of a subset of the bHLH transcription factor superfamily. It has been reported that apples have 188 *MdbHLH* genes [32]. To identify the *PIF* members in apples, we utilized the *Arabidopsis* PIF protein sequences to perform a BLASTp analysis, and eight candidate MdPIFs (named MdPIF1 to MdPIF8) were obtained from apples. Depending on the presence of conserved bHLH and APB domains, seven MdPIF proteins were finally determined, and MdPIF6 (GenBank accession number: MDP0000215587) was excluded due to the absence of the APB domain (Table 1 and Figure S1). Sequence analysis revealed that these MdPIF proteins varied widely in length, from 449 amino acid (aa) to 1040 aa, and their molecular mass ranged from 48 KDa to 113 KDa. Other characteristics, including gene IDs, genomic positions, grand average of hydropathicity, and pI values, of the MdPIF proteins are summarized in Table 1.

**Figure 1:**
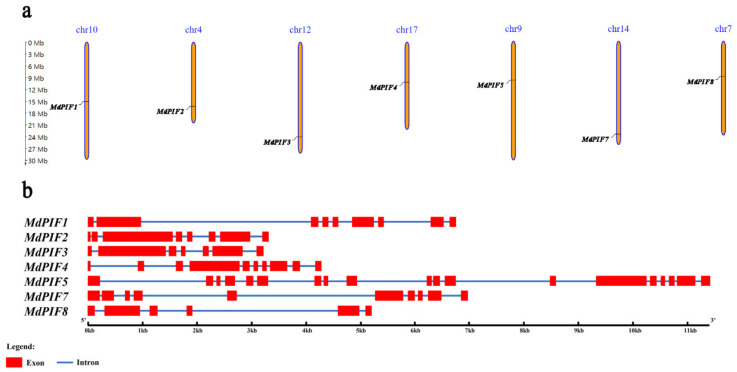
Chromosomal location and gene structure (intron/exon) of the *MdPIF* genes. (**a**) Chromosomal location of seven *MdPIF* genes on seven apple chromosomes. (**b**) Gene structures of the *MdPIF* genes. Red boxes indicate exons and blue lines indicate introns.

Based on the genomic location information obtained from the Genome Database for Rosaceae (GDR), the *MdPIF* genes were mapped onto seven apple chromosomes (Chr): Chr 4, Chr 7, Chr 9, Chr 10, Chr 12, Chr 14, and Chr 17, respectively (Figure 1a). By analyzing the genome DNA sequences, we found that the introns of these *MdPIFs* ranged from 5 to 18 in number. *MdPIF5* contained the highest number of introns (18), whereas *MdPIF8* had only five introns. One group of *MdPIF* genes (*MdPIF4* and *7*) contained the same number of introns/exons (Figure 1b). In addition, *MdPIF2* and *MdPIF3*, both of which were located at the same branch in the phylogenetic tree constructed based on the *MdPIF* coding sequences (Figure S2), had similar intron/exon distributions.

### 2.2. Phylogenetic Analysis, Multiple Sequence Alignment, and Prediction of Conserved MdPIF Motifs

**Figure 2:**
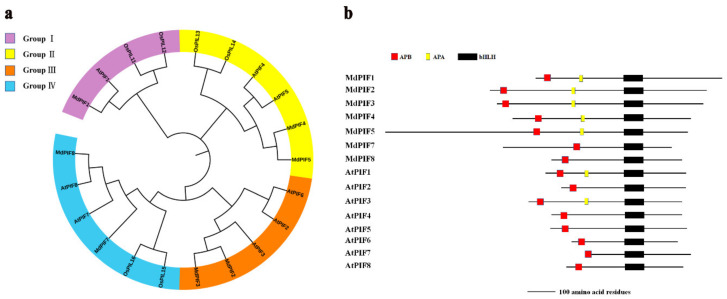
Analysis of phylogenetic relationship and conserved domains of MdPIFs. (**a**) Unrooted phylogenetic tree of PIF proteins from *Malus domestica*, *Arabidopsis thaliana*, and *Oryza sativa.* MEGA5.1 was used to construct the phylogenetic tree based on the PIF protein sequences, and the four distinct subgroups of PIF proteins are shown. Interactive Tree of Life (iTOL) online software was used to annotate and review the phylogenic tree. AtPIF1: AT2G20180; AtPIF2: AT2G46970; AtPIF3: AT1G09530; AtPIF4: AT2G43010; AtPIF5: AT3G59060; AtPIF6: AT3G62090; AtPIF7: AT5G61270; AtPIF8: AT4G00050; OsPIL11: Os12g0610200; OsPIL12: Os03g0639300; OsPIL13: Os03g0782500; OsPIL14: Os07g0143200; OsPIL15: Os01g0286100; OsPIL16: Os05g0139100. (**b**) Motif comparisons of the MdPIF and AtPIF proteins. The presence of APA, APB, and bHLH motif is depicted as boxes. Bar = 100 amino acid residues.

As PIFs are bHLH transcription factors that are conserved among different plant species, we next conducted a phylogenetic analysis based on the protein sequences of PIFs from apple, *Arabidopsis*, and rice (*Oryza sativa*) to investigate their evolutionary relationship. We found that MdPIF proteins were categorized into four groups. MdPIF1 belonged to group I; MdPIF4 and MdPIF5 were in group II; MdPIF2 and MdPIF3 were assigned into group III; and MdPIF7 and MdPIF8 were categorized into group IV (Figure 2a). Moreover, the MdPIFs had a closer relationship with AtPIFs than with OsPIFs, which was probably because both apple and *Arabidopsis* species are dicotyledons, whereas rice species are monocotyledons.

The presence of bHLH and APB domains has been developed as a basic criterion for screening PIF members. Here, the two highly conserved domains were identified in all MdPIF proteins (Figure 2b and Figure S3a). In addition, an APA domain was also found to be present at the N terminal of several MdPIF proteins (MdPIF1, MdPIF2, MdPIF3, MdPIF4, and MdPIF5) (Figure 2b and Figure S3b), which is similar to AtPIF1 and AtPIF3 [8,15].

**Figure 3:**
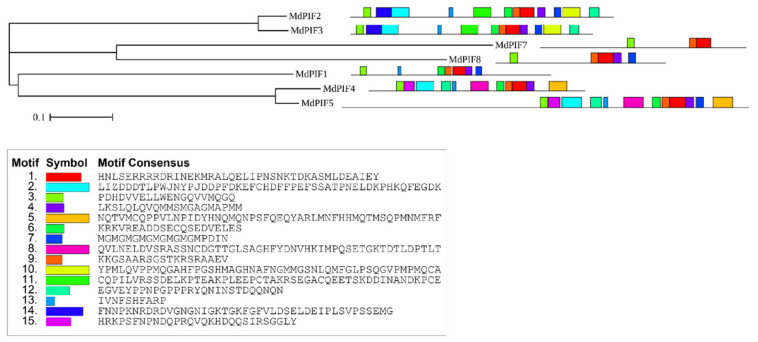
Schematic representation of the conserved motifs predicted in the MdPIF proteins. The MEME program was used to predict the conserved motifs, and TBtools was used to show the results.

Using the MEME motif analysis program, we predicted 15 motifs in the MdPIF proteins (Figure 3). Among them, motif 1 (HLH motif) and motif 3 (APB motif) are widely distributed in all MdPIF proteins. Interestingly, we found that MdPIF members within the same evolutionary branch share a similar motif distribution. For example, MdPIF2 and MdPIF3, both of which are located on the same branch, have highly similar motif composition, and this was also observed for MdPIF4 and MdPIF5 (Figure 3).

### 2.3. Analysis of Cis-Acting Elements in the MdPIF Gene Promoters

PIFs are reported to be involved in plant responses to multiple abiotic factors, and cis-acting elements in the promoter region play a critical role in these processes [8,33,34]. Thus, we next analyzed the cis-acting elements in the promoter regions of *MdPIF* genes to explore their potential regulatory patterns (Figure S4). The results revealed that more than 26 elements are distributed with the promoters of the *MdPIF* genes (Table S2). Based on their function, the cis-acting elements were divided into three groups: light-, hormone-, and stress-responsiveness (Table 2). Among these, light-responsive elements were present in all *MdPIF* gene promoter sequences, including G-box (TACGAT) [35], ACE (CTAACGTATT), LAMP (CTTTATCA), and so on (Table S2). Plant hormonal-responding elements, such as ABRE (ACGTG), GARE (TCTGTTG), and CGTCA, that were involved in responding to ABA, gibberellin (GA), and jasmonic acid (JA), respectively, were also identified in the promoters of *MdPIF* genes (Table S2). In addition, multiple elements responding to abiotic stresses were also found (Table 2 and Table S2). For instance, *MdPIF7*’s promoter contained cis-acting elements responding to low temperature (LTR: CCGAAA) and wounds (WUN-motif: AAATTTCCT). Collectively, the presence of these cis-acting elements indicated that *MdPIF*s might be involved in multiple responses, including responses to light, hormones, and abiotic stresses, which need further efforts to elucidate.

### 2.4. Expression Profiles of the MdPIF Genes

**Figure 4:**
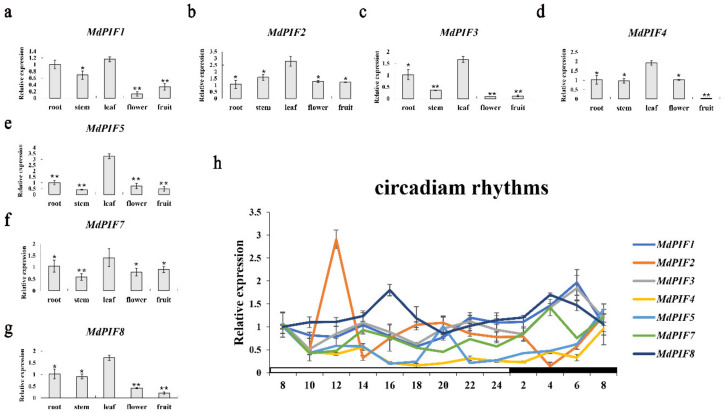
Expression profiles of the *MdPIF* genes. Expression profile of *MdPIF1* (**a**), *MdPIF2* (**b**), *MdPIF3* (**c**), *MdPIF4* (**d**), *MdPIF5* (**e**), *MdPIF7* (**f**), and *MdPIF8* (**g**) in various apple tissues. 18S ribosomal RNA was used as an internal control for qRT-PCR analysis. Data are mean ± SD of three independent biological replicates. Asterisks denote significant differences compared with leaf tissue (*, *p* < 0.05; **, *p* < 0.01). (**h**) Expression profile of the *MdPIFs* in response to day and night. The light conditions are from 8 o’clock to24 o’clock, followed by 8 h of dark environment. Data are mean ± SD of three independent biological replicates. Asterisks denote significant differences from control (*, *p* < 0.05; **, *p* < 0.01).

*PIFs* are important light-responsive genes, therefore we next analyzed the expression profiles of all *MdPIF* genes in response to diurnal rhythm. We first utilized qRT-PCR to determine the expression levels of *MdPIF* genes in different organs, including the root, stem, leaf, flower, and fruit. All the *MdPIF* genes had relatively similar expression patterns. They all had highest expression level in the leaf and the lowest expression level in the flower and fruit (Figure 4a–g), except *MdPIF7* had the lowest expression level in the stem (Figure 4f).

*MdPIF1, 3, 4, 5,* and *7* had similar expression patterns in responding to diurnal rhythm. The transcript levels of the five genes increased significantly overnight and fell dramatically during the day. Among them, *MdPIF5* had a peak change at 8:00 p.m. and its highest level was similar to that observed at dawn. However, the expression level of *MdPIF2* and *MdPIF8* peaked during the day. *MdPIF2* reached the highest level at 12:00 a.m. and then decreased rapidly and gradually accumulated at night. The expression of *MdPIF8* decreased from 4:00 p.m. to 8:00 p.m., and re-accumulated overnight (Figure 4h). Generally, the expression pattern of *MdPIF* genes in response to diurnal cycles was similar to that of *PIF4* and *PIF5* from both *Arabidopsis* and maize, of which the transcript levels increased during the night, peaked at dawn, and dropped during the day [36,37].

### 2.5. Subcellular Localization and Transcriptional Activity Analysis of MdPIFs

**Figure 5:**
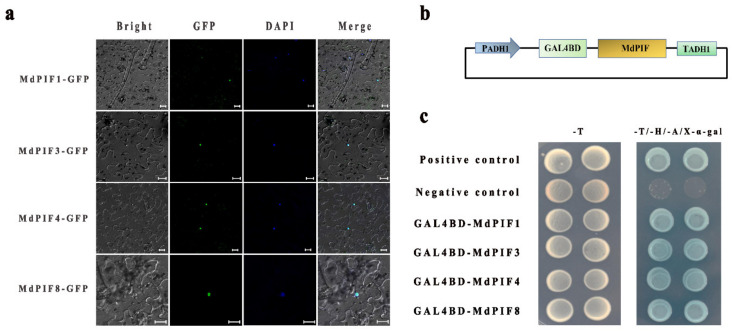
Subcellular localization and transactivation assay of MdPIF proteins. (**a**) Subcellular localization of MdPIF proteins. Scale bar = 20 μm. (**b**) Schematic diagram of the MdPIF-pGBKT7 structure. (**c**) The construct of pGBKT7-MdPIF was transformed into yeast strain AH109 and examined on SD/-Trp and SD/-Trp/-His/-Ade/X-α-gal plates.

Nuclear localization is one of the key common features of transcription factors. Therefore, we next detected the subcellular localization of MdPIF1, MdPIF3, MdPIF4, and MdPIF8, which were selected as representative members of the four groups in the phylogenetic tree (Figure 2a). MdPIF- green fluorescent protein (GFP) constructs were injected into *Nicotiana benthamiana* leaves through agroinfiltration. After two days’ treatment in darkness, the samples were observed under a confocal microscope. All MdPIFs were examined in the nucleus (Figure 5a), which is consistent with the previous reports that PIFs in *Arabidopsis* and maize were all localized in the nucleus [12,38].

The transcriptional activity of MdPIF proteins was also verified in yeast. The coding sequences of *MdPIF* genes were inserted into the pGBKT7 vector (Figure 5b). The empty pGBKT7 vector and pGBKT7-MdMYB23 served as the negative and positive controls, respectively. All of the yeasts within different constructs grew normally on the SD/-Trp medium (Figure 5c). After being transferred to SD/-Trp/-His/-Ade medium supplemented with X-α-gal, blue yeast colonies were present when transformed within pGBKT7-MdPIFs or pGBKT7-MdMYB23, whereas the negative control showed nothing. These results indicated that MdPIF proteins were characterized with transcriptional activity in yeast cells.

### 2.6. Expression Analysis of MdPIFs in Response to Abiotic Stresses and Hormones

Based on the analysis of cis-acting elements in the promoters of *MdPIF* genes (Table 2 and Table S2), it was reasonable to hypothesize that *MdPIFs* were involved in responding to abiotic factors and plant hormones. To verify our hypothesis, we next investigated the expression patterns of the four representative *MdPIF* genes (*MdPIF1*, *MdPIF3*, *MdPIF4*, and *MdPIF8*) after being treated by 100 mM NaCl (which mimicked salt stress), 8% PEG 6000 (which mimicked drought stress), 100 μM indole-3-acetic acid (IAA), 100 μM ABA, and 100 μM GA, respectively.

**Figure 6:**
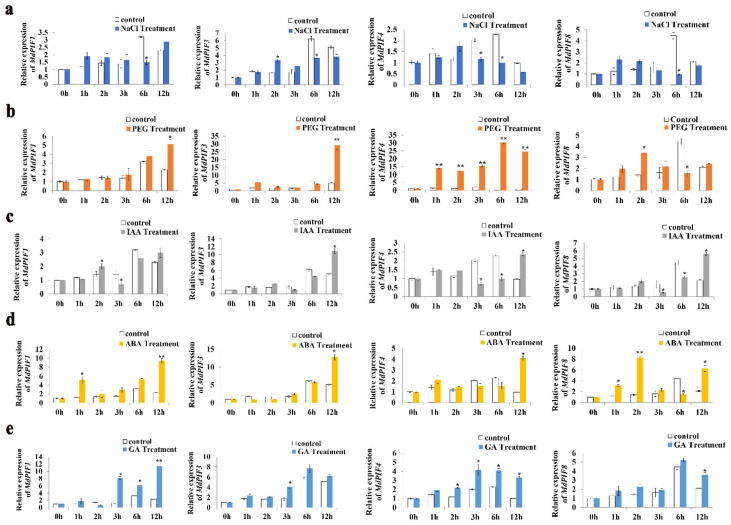
Expression analysis of *MdPIF* genes in response to abiotic stressors and hormones. Expression levels of the *MdPIFs* in response to NaCl (**a**), PEG 6000 (**b**), indole-3-acetic acid (IAA) (**c**), ABA (**d**)**,** and GA (**e**). Data are mean ± SD of three independent replicates. Asterisks denote significant differences from control (*, *p* < 0.05; **, *p* < 0.01).

As shown in Figure 6, the expression level of *MdPIF* genes in the treatment groups indicated the responsiveness of *MdPIFs* to several abiotic stresses and hormones, despite the fluctuating expression of *MdPIFs* in the control group. Specifically, under NaCl treatment, the expression levels of all *MdPIF* genes were significantly decreased at the 6-h point compared to that of the control, suggesting that salt stress repressed transcription of *MdPIF* genes and that the inhibited expression of *MdPIFs* may be involved in resistance to salt stress (Figure 6a). Furthermore, when treated with PEG 6000, expression levels of *MdPIF1* and *MdPIF3* increased significantly after 12 h, whereas those of *MdPIF4* and *MdPIF8* were upregulated dramatically at an early stage (the 1-h and 2-h points, respectively). However, when expression levels of *MdPIF8* decreased after the peak (2 h), *MdPIF4* expression levels remained at high levels and peaked at 6 h (Figure 6b).

In addition to responding to abiotic stresses, the expression levels of *MdPIF* genes were also influenced by exogenous hormones to vary degrees. For instance, under IAA treatment, expression levels of all *MdPIF* genes were downregulated after 3 h, and then gradually increased from 6 h to 12 h (Figure 6c). When treated with ABA, *MdPIF3* and *MdPIF4* were all expressed highly after 12 h compared to controls, whereas *MdPIF1* and *MdPIF8* expression levels were upregulated at both 1 h and 12 h (Figure 6d). Furthermore, *MdPIF1, MdPIF3,* and *MdPIF4* showed significantly increased transcript levels 3 h post-treatment with GA, whereas *MdPIF8* was expressed highly after 12 h (Figure 6e). Collectively, these data suggest that the apple *MdPIF* genes were widely involved in multiple abiotic stress and hormones responses.

### 2.7. MdPIF4 Overexpression Reduces Sensitivity to ABA in Apple Calluses

**Figure 7:**
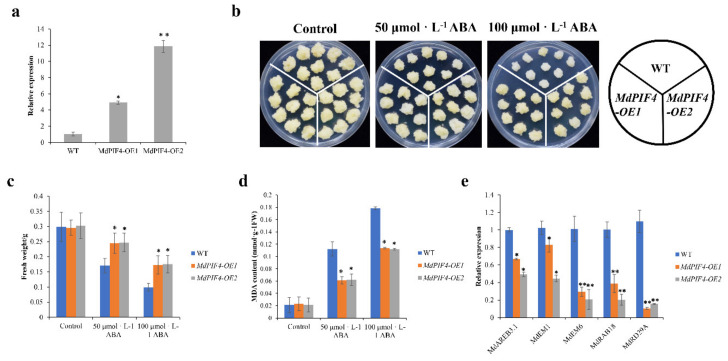
Reduced ABA sensitivity of *MdPIF4* transgenic apple callus. (**a**) Expression analysis of *MdPIF4* in WT and transgenic calluses. 18S ribosomal RNA was used as an internal control. (**b**) The phenotypes of 14-day-old transgenic and WT apple calluses cultured on Murashige and Skoog (MS) medium containing different concentrations of ABA for 20 d. (**c**) Statistical analysis of fresh weights for the transgenic and WT apple calluses after treatment. (**d**) Statistical analysis of the MDA concentrations in transgenic and WT apple calluses after treatment. (**e**) The gene expression of ABA response genes in WT and transgenic calluses after ABA treatment. Data are mean ± SD of three independent replicates. Asterisks denote significant differences from control (*, *p* < 0.05; **, *p* < 0.01).

Among the seven *MdPIF* gene promoters, a large number of ABA-responsive elements were found, indicating these apple *PIF* genes might be involved in the ABA response. The promoter of *MdPIF4* contained the largest number of ABA-responsive elements (Table 2), and the expression level of *MdPIF4* was significantly influenced by ABA treatment (Figure 6d). We therefore predicted that *MdPIF4* was of highly likely to be involved in ABA signaling pathways. We first obtained two transgenic lines of apple calluses with overexpressed *MdPIF4*. Transgenic calluses generated much higher transcription levels of *MdPIF4* compared to the WT control, suggesting that *MdPIF4* was successfully transformedinto the callus (Figure 7a). In mock treatment, the growth of the WT and the *MdPIF4-OE* callus were consistent, and they had similar fresh weights, as well as MDA content, which is the most mutagenic product of lipid peroxidation and its content has been developed to be an important index for measuring the degree of plant damage caused by stresses [39,40,41] (Figure 7b,d). Exogenous ABA treatment restricts cell elongation and thus inhibits apple callus growth, which has been demonstrated by previous reports [42]. When supplied with ABA in the medium, the growth of WT and *MdPIF4*-OE calluses was inhibited compared to that in mock conditions, and higher ABA concentrations made it worse (Figure 7b). Nevertheless, the *MdPIF4*-OE callus grew much better than WT, suggesting that overexpressed *MdPIF4* in apple calluses reduces their sensitivity to ABA (Figure 7b). The fresh weights of apple calluses with overexpressed *MdPIF4* were significantly higher than that of WT under ABA treatment (Figure 7c), which was consistent with the phenotypic results displayed in Figure 7b. Moreover, the *MdPIF4-OE* callus had a lower MDA content compared to that of WT, suggesting that overexpressed *MdPIF4* alleviated the damage caused by ABA and thus promoted the callus growth more effectively under ABA treatment.

To further investigate the role of *MdPIF4* in regulating ABA response, we tested the expression pattern of several ABA response genes in WT and transgenic apple calluses. ABA response genes, such as *MdEM1*, *MdEM6*, *MdRAB18*, and *MdRD29A*, that were reported to be involved in ABA downstream signaling pathways, as well as *MdAREB3.1,* which regulates ABA signaling pathways [43], were significantly downregulated in the *MdPIF4*-*OE* callus compared to that in WT apples (Figure 7e), suggesting that *MdPIF4* might be involved in modulating the expression of ABA response genes to regulate ABA sensitivity.

## 3. Discussion

With the completion of whole genome sequencing of apples [44,45,46], many transcription factor families have been identified, such as MYB [47], WRKY [48], BBX [49], and bHLH [32]. Some of the pivotal genes have been characterized and their biological functions have been widely studied, such as *MYB1* [50], *WRKY40* [51], and *BBX22* [52]. Although *PIF*s play an important role in plant growth and development, systematical characterization and functional investigation have not been conducted with the *PIF* family in apple, except with *MdPIF1* [31]. Here, a systematic analysis of the *MdPIF* gene family was performed, which provides directions for further research on the *PIF* gene in apples and in the selection of important trait genes.

### 3.1. PIFs Are Transcription Factors Conserved among Different Plant Species

In this study, seven *MdPIF* genes were identified in apples, which is similar to the numbers reported in other plants—eight for *Arabidopsis* [9], six for rice [53], and seven for maize [38]. Phylogenetic analysis showed that MdPIFs were divided into four clades (Figure 2a). The results are different from previous reports on the evolutionary relationship of PIF proteins in maize and rice, in which PIF proteins are clustered into three clades [38,53]. The analysis of MdPIF protein sequences showed that all MdPIFs share the conserved bHLH domain and APB motif. Five MdPIFs (MdPIF1, MdPIF2, MdPIF3, MdPIF4, and MdPIF5) have the APA motif, similar to AtPIF1 and AtPIF3, suggesting that these MdPIFs might be close to AtPIF1 and AtPIF3 on an evolutionary scale (Figure 2b). Moreover, it is worth mentioning that MdPIF7 and MdPIF8 display an amino acid substitution in the APB domain that alters the conserved Q residue to X and E, respectively (Figure S3a) [14]. Meanwhile, additions of amino acid residue in bHLH domain were specific in MdPIF1 and MdPIF2 compared to other MdPIFs, suggesting that they may have a potentially unique function (Figure S3a). Expression pattern analysis in different apple tissues found that all *MdPIF* genes were constitutively expressed in the tissues examined, with relatively high levels in leaves (Figure 4a–g), which is similar to the expression pattern of *AtPIFs* and *ZmPIFs* [38,54]. The nuclear localization (Figure 5a) and transcriptional activation activity (Figure 5c) of MdPIFs indicated that they are typical transcription factors, and that they may function as positive regulators of downstream gene expression.

### 3.2. PIFs Act as a Molecular Hub in Integrating Environmental and Hormonal Signaling Pathways

In addition to being negative regulators of photomorphogensis, a growing body of evidence indicates that PIFs act as a signaling hub and play key roles in numerous processes, including anthocyanin synthesis [18]; resistance to drought [22,23], salt [23], and cold [55]; signaling pathways of plant hormones (GA, BR, and auxin) [56]; and even in regulating plant immunity [57].

The cis-acting elements in the gene promoter region can be recognized by multiple cellular proteins, such as transcription factors, to regulate transcription and gene expression in response to environmental and hormonal signals. For example, *AtPIF4* mRNA expression is activated by high temperature. It was further found that high temperature inactivates AtELF3 (early flowering 3), which represses *AtPIF4* expression by directly binding to its promoter [56]. Moreover, the binding affinity of AtELF3 to the *AtPIF4* promoter is decreased at a high temperature (27 °C), compared to that at a normal temperature (22 °C) [58]. When analyzing the promoter of *MdPIF* genes, light-responsive related elements appear to be significantly abundant (Table 2 and Table S2). Combining the changes in the transcription level of *MdPIF* genes in day and night in Figure 4h, we found that *MdPIF* genes were regulated by a light-mediated diurnal rhythm. In addition to the photoperiod, multiple cis-acting elements were predicted to respond to plant hormones (ABA, GA, and JA) and abiotic stresses (drought, low temperature, and wound) (Table 2 and Table S2). Previous studies have shown that some hormones, such as ABA, GA, and JA, play a key role in regulating plant responses to stress conditions [59]. Our data also confirmed that *MdPIF* expression could be induced by several abiotic stresses and hormones (Figure 6). For example, *MdPIF1* and *MdPIF4* were significantly upregulated by drought and GA treatments (Figure 6b,e). Motifs that were involved in the response to drought (MBS: CAACTG) and ABA (ABRE: ACGTG) were predicted to be present in *MdPIF3*’s promoter (Table 2 and Table S2), and the expression level of *MdPIF3* was significantly induced after drought and ABA treatment for 12h (Figure 6b,d). In addition, cis elements that function in response to GA were present in the promoter of *MdPIF3* and *MdPIF4*, and both of them were induced to express to varying degrees by GA treatment (Figure 6e). Moreover, ABA responsive motifs were found in the promoters of most *MdPIF* genes, and these genes were induced to varying degrees under ABA treatment (Figure 6d). These data suggested that the cis elements in the promoter region of *MdPIFs* may be functional in their response to abiotic stresses and hormones.

A growing body of evidence indicates that *PIF4* acts as a key integrator of multiple signaling pathways [56], including light (skomotorphogenesis) and temperature signaling pathways [21], hormonal signaling pathways (auxin, GA, and BR), and circadian clock output pathways. Here, we found that ABA treatment affected the *MdPIF4* expression (Figure 6d), and overexpressed *MdPIF4* decreased the sensitivity of apple calluses to ABA by downregulating the expression of ABA-responsive genes (Figure 7), indicating that *MdPIF4* might play a negative regulatory role in ABA signaling pathways, which has not been reported yet, and the mechanism behind this still needs further investigation. In addition, several genes involved in ABA response have been identified in apples. The expression of seven *MdAGO* genes from the *MdAGO* family were significantly enhanced by ABA treatment [60]. Overexpression of *MdSINA2* increased ABA sensitivity in apple calluses and *Arabidopsis* [61]. Although the regulatory mechanism remains to be resolved, the identification of these ABA-responsive genes has enriched our knowledge of the ABA signaling pathway network in apples, and will provide a basis for future research on their functions.

## 4. Materials and Methods

### 4.1. Plant Materials and Growth Conditions

Apple tissue (*Malus domestica* ‘Royal Gala’) samples (stem, young root, mature leaf, flower, and ripening fruit) were collected from three 10-year-old apple trees in an artificially managed orchard to investigate expression pattern of the *MdPIF*s [62,63]. Of note, ripening fruits were collected 150 days after bloom and roots were taken from young roots that were newly grown in the year. Tissue-cultured apple (*Malus domestica* ‘Royal Gala’) seedlings were maintained on Murashige and Skoog (MS) medium with 0.5 mM 6-benzylaminopurine (6-BA) and 0.1 mM indole-3-acetic acid (IAA) during a 16 h/8 h of light/dark photoperiod (light intensity 70 μmol m^−2^ s^−1^) in a culture room at 25 °C [32]. After 21 d of growth, the seedlings were transferred to root-inducing medium (MS medium within 0.1 mM IAA) to induce root growth [64]. These tissue-cultured apple seedlings were used to measure diurnal expression at 8:00, 10:00, 12:00, 14:00, 16:00, 18:00, 20:00, 22:00, 24:00, 2:00^2d^ (second day), 4:00^2d^, 6:00^2d^, and 8:00^2d^. The light started from 8 o’clock and ended at 24 o’clock, followed by 8 h of darkness. For abiotic stresses treatment, the untreated apple seedlings were transferred to water-cultured conditions. Under normal photoperiod conditions, the seedlings with the same growth state were treated in NaCl (100 mM), polyethylene glycol 6000 (PEG 6000, 8%), IAA (100 μM), ABA (100 μM), and gibberellin (GA) (100 μM), respectively [65,66]. These seedlings were collected at 0 h, 1 h, 2 h, 3 h, 6 h, and 12 h after treatment, immediately frozen by liquid nitrogen, and stored at −80 °C for further analysis [67].

The ‘Orin’ apple (*Golden Delicious* × *Indo*) calluses were cultured on MS medium containing 1.5 mg/L 2,4-dichlorophenoxyacetic acid (2, 4-D) and 0.4 mg/L 6-BA at 24 °C for 20 d in the dark [52].

### 4.2. Plasmid Construction and Acquisition of the Transgenic Plant Material

The full-length cDNA of *MdPIF4* was cloned into the pRI 101-AN vector (Takara, Dalian, China) to obtain the *MdPIF4* overexpression construct. Then, the pRI 101-AN empty vector and recombinant construct were transformed into *Agrobacterium tumefaciens* strain LBA4404. These agrobacteria were grown in lysogeny broth (LB) medium supplemented with 50 mg/mL kanamycin and 50 mg/mL rifampicin [68].

Transgenic apple calluses were obtained by agrobacterium-mediated transformation [69]. Briefly, the 14-day-old apple calluses were co-cultured with agrobacterium strains carrying the empty vector or recombinant constructs in the dark for 20 min. Then the transformed apple calluses were plated on selective medium supplemented with 250 mg/L carbenicillin and 30 mg/L kanamycin [70]. The successfully transgenic apple calluses grew in about four weeks.

### 4.3. Identification, Chromosomal Location, and Functional Annotation of the MdPIF Genes

To identify the *MdPIF* genes from the *Malus domestica* genome, we downloaded eight PIF peptide sequences from the *Arabidopsis* database [71]. Then, MdPIFs were searched in the apple genome database (*Malus domestica* v1.0) using the *Arabidopsis* protein sequences [72]. Eight candidate protein sequences were screened out, and we next used the SMART database [73] to check whether these candidates contained the conserved bHLH and APB domains.

The chromosomal location of these genes were found in the apple genome annotation file (*Malus domestica*.v1.0.consensus.gff) from the Genome Database for Rosaceae (GDR) [74]. The *MdPIF* genes were submitted to online MG2C software [75] for gene chromosome mapping. The molecular mass, theoretical isoelectric point (pI), and protein hydrophobicity of these MdPIFs were analyzed using the online tool ProtParam [76].

### 4.4. Phylogenetic Analysis of the PIF Proteins

AtPIF protein sequences were obtained from the *Arabidopsis* database [71,77]. The PIF protein sequences of rice were obtained from the rice annotation project [78]. Based on all PIF protein sequences from *Arabidopsis*, rice, and apple, a phylogenetic tree was constructed using MEGA5.1 software via the neighbor-joining method with the following settings: Bootstrap method, 1000 replicates, and the Poisson model [79,80].

### 4.5. Gene Structure (Intron/Exon), Multiple Sequence Alignment, and Conserved Motif Analysis

The apple genome database file (*Malus domestica*.v1.0.consensus.gff) was downloaded from the GDR database. Structure information of the *MdPIF* genes was drawn using the gene feature visualization server GSDS 2.0 [81,82,83]. The sequence alignment was performed using DANMAN software with default parameters [42,84]. The conserved motif analysis was carried out using the MEME program [85], and TBtools was used to display the motif structure [86].

### 4.6. Analysis of Cis-Acting Elements in the MdPIF Promoters

A 1500-bp fragment at the upstream of the start codon of the *MdPIF* genes was considered the promoter region, and the promoter sequences were submitted to PlantCARE [87] to analyze the cis-acting elements [88,89].

### 4.7. RNA Extraction and Quantitative Real-Time PCR (qRT-PCR) Analysis

Total RNAs were extracted with the RNAplant Plus Reagent Kit (Tiangen, Beijing, China). Reverse transcription was performed using the PrimeScript™ RT Reagent Kit (Takara, Shiga, Japan). The qRT-PCR reaction system contained 10 μL SYBR premix Ex Taq™ (Takara), 0.8 µL (10 mΜ) of each primer, and 50 ng of cDNA, in a total volume of 20 μL. The qRT-PCR reaction procedure was performed according to methods previously reported [90]. The PCR experimental sample had three repetitions and 18S ribosomal RNA served as a control [91]. The 2^−ΔΔCT^ 2^−ΔΔCT^ method was used for data analysis. All primers used in this study are shown in Table S1.

### 4.8. Subcellular Localization Analysis

The coding sequences of *MdPIF1*, *MdPIF3*, *MdPIF4,* and *MdPIF8* were cloned into a *35S::GFP* vector for translational fusion with the green fluorescent protein (GFP), respectively. The recombinant expression vectors *35S::MdPIF-GFP* were transformed into the *Agrobacterium* LBA4400 strain, respectively. Then, the agrobacterium-harboring *35S::MdPIF-GFP* constructs were injected into *Nicotiana benthamiana* leaves [92] and cultured in the dark for 2 days. GFP signal in cells was observed using a high-resolution confocal microscope (LSCM Meta; Carl Zeiss, Zena, Germany) [93].

### 4.9. Transcriptional Activity Assay

The coding sequences of *MdPIF1*, *MdPIF3*, *MdPIF4,* and *MdPIF8* were cloned into the pGBKT7 vector using the homologous recombination method. Restriction endonuclease sites were EcoRI and SalI (Table S1). Since MdMYB23 (GenBank accession number: MDP0000230141) has been proven to have transcriptional activity in our laboratory [94], it was able to be used as a positive control. The pGBKT7-MdPIF fusion vectors, employing the pGBKT7 vector (negative control), and pGBKT7-MdMYB23 (positive control), were transferred into the yeast strain AH109. Then they were cultured on the SD medium (SD/-Trp, SD/-Trp/-His/-Ade/X-α-gal) at 28 °C for 3–5 days.

### 4.10. Apple Callus Treatment and Physiological Measurements

The 14-day old WT and transgenic apple calluses were cultured on MS medium containing 0 μM, 50 μM, and 100 μM ABA. An electronic balance (one thousandth) was used to measure the weight of apple calluses to determine the callus growth. Malondialdehyde (MDA) content was measured using the thiobarbituric acid method reported previously [43].

### 4.11. Statistical Analysis

All data were obtained from three independent experiments with three biological duplicates in each experiment. DPS software was used for significant difference analysis, and a *p*-value < 0.05 was considered a significant difference [95]. Error bars represent the standard deviation.

## 5. Conclusions

We screened out seven *MdPIF* genes from the apple genome and four of them were selected as representative members for further investigation. We systematically analyzed the bioinformatic features of the *MdPIF*s and found that their expression could be affected by multiple abiotic stresses and hormones. Subcellular localization and transcriptional activity analysis showed that MdPIFs were typical nuclear transcription factors with transcriptional activation ability. Through analysis of transgenic apple calluses, we found overexpressed *MdPIF4* in apple calluses reduces their sensitivity to ABA. Our data improved our understanding of the *MdPIFs’* functions, and also sheds light for future exploration of PIFs’ critical roles in multiple biological processes in apples and other plant species.

## Figures and Tables

**Table 1:** Information about the *PIF* members found in apples.

Gene Name	Gene ID	Chromosome Location	Position	mRNA Length (bp)	CDS Length (bp)	Amino Acid Length (aa)	Molecular Weight (Da)	TheoreticalpI	Grand Average of Hydropathicity (GRAVY)	Best Hits
*MdPIF1*	MDP0000289642	chr10	16985872-16992614	6743	1920	639	70621.62	8.55	−0.59	*AtPIF1*
*MdPIF2*	MDP0000205358	chr4	18339668-18342979	3312	2235	744	80495.75	6.52	−0.645	*AtPIF2*
*MdPIF3*	MDP0000290263	chr12	27035201-27038407	3207	2127	708	75773.44	5.83	−0.626	*AtPIF3*
*MdPIF4*	MDP0000198404	chr17	11357450-11361687	4238	1842	613	67336.57	7.06	−0.676	*AtPIF4*
*MdPIF5*	MDP0000254650	chr9	11107477-11118877	11401	3123	1040	113679.29	8.76	−0.52	*AtPIF5*
*MdPIF7*	MDP0000319248	chr14	26344135-26351096	6962	1746	581	64713.13	9.96	−0.903	*AtPIF7*
*MdPIF8*	MDP0000439540	chr7	10087113-10092308	5196	1350	449	48033.51	7.72	−0.445	*AtPIF8*

**Table 2:** Cis-acting element analysis in the *MdPIF* gene promoters.

Gene	Light Response			Hormone Response					Stress Response		
		AUX	ABA	GA	JA	SA	DT	LT	DnS	WD	AAI
*MdPIF1*	4/1				1/1	1/0		0/1			
*MdPIF2*	8/5		1/2		1/1		0/1		1/0		1/0
*MdPIF3*	7/4		0/2	1/0			1/1		0/1	0/1	2/1
*MdPIF4*	8/5	1/0	3/2	1/0	1/1						0/1
*MdPIF5*	6/6		1/1		2/0		1/0		1/0		1/3
*MdPIF7*	6/3		1/1	1/2				0/1		1/1	2/1
*MdPIF8*	7/2		0/1						1/0		1/1

AUX, auxin; ABA, abscisic acid; GA, gibberellin; JA, MeJA; SA, salicylic acid; DT, drought; LT, low temperature; Dns, defense and stress; WD, wound; AAI, anaerobic induction. The numbers in the table represent the number of cis-acting elements on the positive and negative chains.

## Supplementary Materials

Supplementary materials can be found at https://www.mdpi.com/1422-0067/21/19/7350/s1.

## Abbreviations

bHLH	Basic helix-loop-helix
APB	Active phyB-binding
APA	Active phyA-binding
ABA	Abscisic acid
