# Supplementary material for: Identification of Phytochrome-Interacting Factor Family Members and Functional Analysis of MdPIF4 in Malus domestica
Source: Int J Mol Sci. 2020 Oct 5;21(19):7350. doi: 10.3390/ijms21197350 (PMC7582839; doi:10.3390/ijms21197350)
Supplement: Supplementary file 1 [file ijms-21-07350-s001.pdf]

Supplementary Materials

>MDP0000215587

MGDTYECDHYYKNATCSSSPPAAPPHSAADDMSLFLQQILVRSSTSLASGKAPQSLFSSSPSVGALLPGNL
DRPCHSGFLGDGIPAVDSFAAFVSGHPNEASENEADEDDCESEEGLEAFVEARPGGGRSSSKRSRAAEVHN
LSEKRRRSRINEKMKALQNLIPNSNKTDKASMLDEAIEYLKQLQLQVQMLSMRNGMSLHPLYLPGTLQP
VQLSQMRMELGEENRPLHLDMTGTLHMNLESSTQNLFNLSNQCTAANESYVPDMSNVVNSETSFVLEPSI
QSHLGPFQLPASSQEICRDDLLQHQKIDVNHSEMNLSVTATVSLPFAQVSDPKSTFDTCIIGRDRQEVGLLR
SIEQNFHIDRCLGIFELS

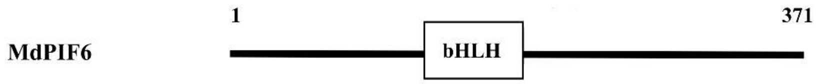

Figure S1. Amino acid sequence of candidate MdPIF6.

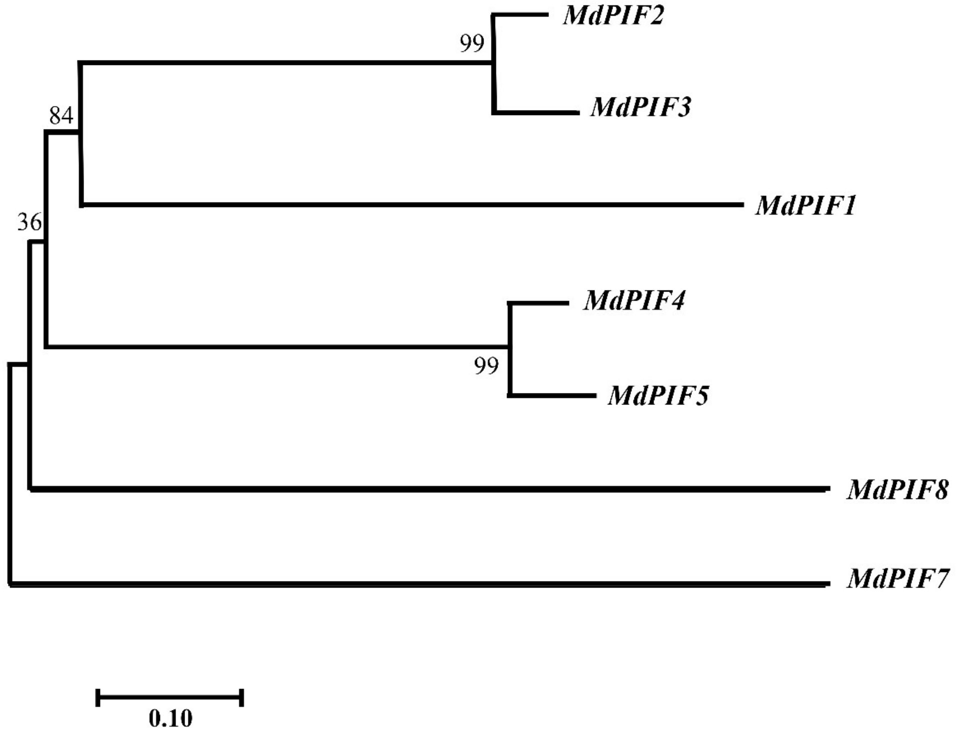

Figure S2. Phylogenetic tree analysis of the *MdPIF* cDNA sequences.

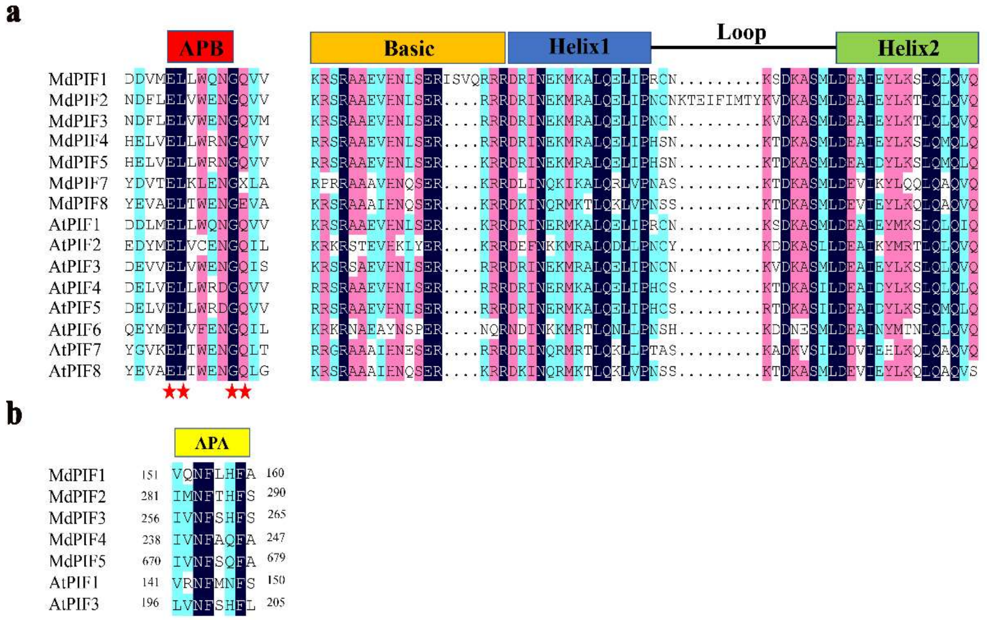

Figure S3. **(a)** Multiple sequence alignment of the bHLH and APB domains of the PIF proteins. Asterisks represent conserved amino acid residues. **(b)** Multiple sequence alignment of the APA domain in the PIF proteins.

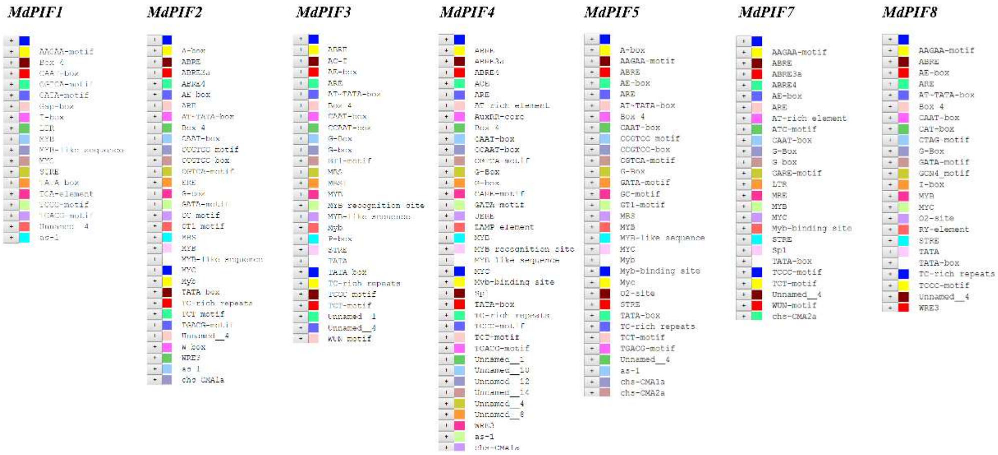

Figure S4. *Cis*-acting element analysis in the *MdPIF* genes promoter regions.

Table S1. Primers used for the gene expression analysis and vector construction in this study.

Primer name	Sequence (5′→3′)
18S-F	ACACGGGGAGGTAGTGACAA
18S-R	CCTCCAATGGATCCTCGTTA
MdPIF1(qRT)-F	ATGAATGGGTGCGTCCCCGATTTCGAA
MdPIF1(qRT)-R	TAGAGGAGGTCCGCGGAGAAGGGGT
MdPIF2(qRT)-F	CAGGAACAATCTGCCCGAGT
MdPIF2(qRT)-R	CTAAGGGCGCACATTGCATC
MdPIF3(qRT)-F	GTCAAGGAGTTCCAGTGCCGATG
MdPIF3(qRT)-R	TCCGCTTGGTGAGTTCATGTTCTG
MdPIF4(qRT)-F	TGGATGGGAAGCGGGATGGC
MdPIF4(qRT)-R	CGAGCGGGTGCCTAGGTAGAC
MdPIF5(qRT)-F	CTAGGCATCCGCTCGTTGAT
MdPIF5(qRT)-R	CAGCTCTTCCACCGAAAGGT
MdPIF7(qRT)-F	TAGTGGATCGGGGGACCATT
MdPIF7(qRT)-R	CATCTCGGTTTTCCAGCCCT
MdPIF8(qRT)-F	TTCTCGCTCCCACTCCCTTCAC
MdPIF8(qRT)-R	GCTACCTCGCCGTTTTCCCATG
MdAREB3.1(qRT)-F	GGTGCTTGCTGGGTATTAATCTC
MdAREB3.1(qRT)-R	TCTCCTCTCTCTCTCTCTAGC
MdEM1(qRT)-F	AATGGGGGAGCTGGATAATTCCT
MdEM1(qRT)-R	CTAGAGCAATGAGACCTATCGGGC
MdEM6(qRT)-F	AATGGGGGAGCTGGATAATTCCT
MdEM6(qRT)-R	CTAGTGCTTCAACTTCGGTTTCAAT
MdRAB18(qRT)-F	ATGGAAACCTCTACCAAGTCTG
MdRAB18(qRT)-R	TTACCGGACCTCGGGCCGAAA
MdRD29A(qRT)-F	ATGGAGAGTGGAGGTTCTGCAAGT
MdRD29A(qRT)-R	CTAGATTGGCTTACATCTTTTGCCT
MdPIF1-F	ATGAATGGGTGCGTCCCCGATTTCG
MdPIF1-R	TCACGTGGTGAGTGCTATATGACGTA
MdPIF3-F	ATGCCTTTGTCCGAGCTTTATCGGATG
MdPIF3-R	TTATCCGTTAGCTCTGRTGTTGTT
MdPIF4-F	ATGGATACCAAGTCTAATGCTTGTA
MdPIF4-R	TCAATTGTCCGGCGTGAGATTCTT
MdPIF8-F	ATGAGCCAGCGCGTTCCTAGCTGGG
MdPIF8-R	TTAGCCCTTAGAGCTAGATGCTGGA
MdPIF1(GFP)-F	AAGGAGCCCTTCACC ATGAATGGGTGCGTCCCCGATTTCG
MdPIF1(GFP)-R	GGCGCGCCCACCCTTTCACGTGGTGAGTGCTATATGACGTA
MdPIF3(GFP)-F	AAGGAGCCCTTCACCATGCCTTTGTCCGAGCTTTATCGGATG
MdPIF3(GFP)-R	GGCGCGCCCACCCTTTTATCCGTTAGCTCTGRTGTTGTT
MdPIF4(GFP)-F	AAGGAGCCCTTCACCATGGATACCAAGTCTAATGCTTGTA
MdPIF4(GFP)-R	GGCGCGCCCACCCTT TCAATTGTCCGGCGTGAGATTCTT
MdPIF8(GFP)-F	AAGGAGCCCTTCACCATGAGCCAGCGCGTTCCTAGCTGGG
MdPIF8(GFP)-R	GGCGCGCCCACCCTT TTAGCCCTTAGAGCTAGATGCTGGA

MdPIF4(OE)-F	GTCGACATGGATACCAAGTCTAATGCTTGTA
MdPIF4(OE)-R	GGATCCATTGTCCGGCGTGAGATTCTTACATT
MdPIF1(GAL4BD)-F	ATGGCCATGGAGGCCGAATTCATGAATGGGTGCGTCCCC
MdPIF1(GAL4BD)-R	ATGCGGCCGCTGCAGGTCGACTCACGTGGTGAGTGCTATATGACG
MdPIF3(GAL4BD)-F	ATGGCCATGGAGGCCGAATTCATGCCTTTGTCCGAGCTTTATC
MdPIF3(GAL4BD)-R	ATGCGGCCGCTGCAGGTCGACTCCGTTAGCTCTGTGTTGTTAACAT
MdPIF4(GAL4BD)-F	ATGGCCATGGAGGCCGAATTCATGGATACCAAGTCTAATGCTTGTATA
MdPIF4(GAL4BD)-R	GA
MdPIF8(GAL4BD)-F	ATGCGGCCGCTGCAGGTCGACATTGTCCGGCGTGAGATTCTT
MdPIF8(GAL4BD)-R	ATGGCCATGGAGGCCGAATTCATGAGCCAGCGCGTTCCT
	ATGGCCATGGAGGCCGAATTCATGAGCCAGCGCGTTCCT

Table S2. *Cis*-acting element analysis in the *MdPIF* genes promoter regions. The numbers in the table represent the number of *cis*-acting elements on the positive and negative chains.

Cis-acting element Name	Signal Sequence	*MdPIF1*	*MdPIF2*	*MdPIF3*	*MdPIF4*	*MdPIF5*	*MdPIF7*	*MdPIF8*
**ATC**	AGTAATCT						1/0	
**LAMP**	CTTTATCA				1/0			
**GATA**	AAGGATAAGG	0/1	1/0		0/1	0/1		0/1
**ACE**	CTAACGTATT				1/0			
**TCT-motif**	TCTTAC		0/1	0/2	1/0		1/0	
**TCCC-motif**	TCTCCCT	0/1			0/1		1/0	1/0
**AE-box**	AGAAACAA		0/1	1/1		0/1	1/1	2/0
**G-box**	TACGTG		3/1	2/0	4/2	1/1	2/1	1/0
**GT1-box**	GGTTAA		1/0	1/1		0/2		
**Box4**	ATTAAT	0/1	3/1	2/0	1/0	3/1		3/1
**chs-CMA1a**	TTACTTAA		0/1		0/1	1/0		
**chs-CMA2a**	TCACTTGA					1/0	0/1	
**Gap-box**	CAAATGAA(A/G)A	1/0						
**I-box**	AGATAAGG	0/1						
**AUXBR-core**	GGTCCAT				1/0			
**ABRE**	ACGTG		1/2	0/2	3/2	1/1	1/1	0/1
**GARE**	TCTGTTG			1/0	1/0		1/2	
**CGTCA**	CGTCA	1/0	1/0			2/0		
**TGACG**	TGACG	0/1	0/1		1/0			
**TCA-element**	CCATCTTTTT	1/0						
**P-box**	CCTTTTG			1/0				

**MBS**	CAACTG		0/1	1/1		1/0		
**LTR**	CCGAAA	0/1					0/1	
**TC-rich**	ATTCTCTAAC		1/0	0/1		1/0		1/0
**WUN-motif**	AAATTTCCT			0/1			1/1	
**ARE**	AAACCA		1/0	2/1	0/1	1/3	2/1	1/1
